# Safety and efficiency of gasless laparoscopy: a systematic review protocol

**DOI:** 10.1186/s13643-020-01365-y

**Published:** 2020-04-30

**Authors:** Haitham Shoman, Simone Sandler, Alexander Peters, Ameer Farooq, Magdalen Gruendl, Shauna Trinh, James Little, Alex Woods, William Bolton, Abubakar Abioye, David Ljungman

**Affiliations:** 1 Program in Global Surgery and Social Change, Harvard Medical School, Boston, MA USA; 2 Department of Surgery, Weill Cornell Medical College, New York, USA; 3 Division of General Surgery, Department of Surgery, University of Calgary, Calgary, AB Canada; 4 Department of Epidemiology, Technical University Munich, Munich, Germany; 5 Department of Surgery, Riverside University Health System-Medical Center, Moreno Valley, CA USA; 6 Harvard T.H. Chan School of Public Health, Boston, MA USA; 7 Geisel School of Medicine at Dartmouth, Hanover, NH USA; 8 Leeds Institute of Medical Research, University of Leeds, Leeds, UK; 9 Department of Surgery, Institute of Clinical Sciences, Sahlgrenska Academy, University of Gothenburg, Gothenburg, Sweden

**Keywords:** Gasless laparoscopy, Laparotomy, Safety, Efficiency, Insufflation, Pneumoperitoneum, Minimally invasive surgery, Abdominal wall

## Abstract

**Background:**

Gasless laparoscopy, developed in the early 1990s, was a means to minimize the clinical and financial challenges of pneumoperitoneum and general anaesthesia. It has been used in a variety of procedures such as in general surgery and gynecology procedures including diagnostic laparoscopy. There has been increasing evidence of the utility of gasless laparoscopy in resource limited settings where diagnostic imaging is not available. In addition, it may help save costs for hospitals. The aim of this study is to conduct a systematic review of the available evidence surrounding the safety and efficiency of gasless laparoscopy compared to conventional laparoscopy and open techniques and to analyze the benefits that gasless laparoscopy has for low resource setting hospitals.

**Methods:**

This protocol is developed by following the Preferred Reporting Items for Systematic review and Meta-Analysis–Protocols (PRISMA-P). The PRISMA statement guidelines and flowchart will be used to conduct the study itself. MEDLINE (Ovid), Embase, Web of Science, Cochrane Central, and Global Index Medicus (WHO) will be searched and the National Institutes of Health Clinical Trials database. The articles that will be found will be pooled into Covidence article manager software where all the records will be screened for eligibility and duplicates removed. A data extraction spreadsheet will be developed based on variables of interest set a priori. Reviewers will then screen all included studies based on the eligibility criteria. The GRADE tool will be used to assess the quality of the studies and the risk of bias in all the studies will be assessed using the Cochrane Risk assessment tool. The RoB II tool will assed the risk of bias in randomized control studies and the ROBINS I will be used for the non-randomized studies.

**Discussion:**

This study will be a comprehensive review on all published articles found using this search strategy on the safety and efficiency of the use of gasless laparoscopy. The systematic review outcomes will include safety and efficiency of gasless laparoscopy compared to the use of conventional laparoscopy or laparotomy.

**Trial registration:**

The study has been registered in PROSPERO under registration number: CRD42017078338

## Background

Approximately 5 billion people around the world do not have access to safe, timely, and affordable surgical and anesthestic care [1]. Abdominal surgical conditions comprise a burden of disease that cannot be calculated, according to the World Bank’s DCP-3, due to a lack of data [2]. However, the Lancet report on global burden of disease 2013 estimates the global mortality rate per 100,000 for appendicitis is 0.7, gallbladder, and liver conditions is 1.6 and intestinal obstruction is 3.9 [3]. In addition, in India, a study showed that there are 1.1% of deaths (0–69 years) from acute abdominal condition corresponding around 72,000 deaths nationally in 2020 [4]. In high-income settings, many patients requiring abdominal surgery will undergo minimally invasive laparoscopic surgery, prudently preceded by diagnostic imaging. Conventional laparoscopy has a variety of benefits over open laparotomy [5] and can reduce pain [6], reduce wound infection [7], promote faster post-operative recovery [8], and return to quality of life [9].

In resource-limited healthcare settings in low-middle income countries, many patients who require abdominal surgery undergo open laparotomy or go without surgery altogether. Anecdotally, many abdominal conditions remain undiagnosed due to lack of access to diagnostic imaging and diagnostic procedures (i.e., endoscopy, laparoscopy). These resource deficiencies lead to avoidable morbidity and mortality which could be reduced with access to laparoscopic surgery. However, for resource-limited hospitals, conventional laparoscopy is oftentimes unaffordable. Air-tight trochars are expensive, carbon dioxide gas cannot often easily be acquired, and general anaesthesia which is required for patients undergoing pneumoperitoneum, is often impossible without anaesthesiologists and ventilator equipment.

Gasless laparoscopy was developed in the early 1990s [10] to overcome clinical and financial challenges of pneumoperitoneum and general anesthesia. The technique involves a form of mechanical elevation of the anterior abdominal wall. Through either a single incision or multiple ports, a variety of gynaecological [11–14], upper gastrointestinal [15–17], lower gastrointestinal [18–21], and exploratory diagnostic [22, 23] procedures can be performed. Many trials have been conducted investigating the safety outcomes (peri and post-operative complication rate) [21, 24–26] and efficiency outcomes [14, 27] when compared with the mainstay treatment methods.

There are a variety of tested benefits for implementing gasless laparoscopy technique. It may carry significantly reduced costs when compared with conventional laparoscopy, including significantly cheaper equipment costs [21, 26–28] maintenance costs, and anesthesia requirements [21]. Gasless laparoscopy may also have an equal or shorter duration of inpatient stay compared with laparotomy or conventional laparoscopy [11, 22, 28]. Additionally, gasless technique avoids the hemodynamic burden with abdominal insufflation [19, 29, 30], and therefore can be used under spinal anesthesia in patients with hemodynamic compromise (e.g., in exploratory, laparoscopy for abdominal trauma).

For resource-limited hospitals where diagnostic imaging is unavailable, gasless laparoscopy is a financially affordable diagnostic option. Other unstudied benefits may include improved safety outcomes in pregnancy [31], reduced rates of post-site metastases [32], and reduced contamination in contaminated procedures [33]. A common documented risk involved in gasless laparoscopy includes poor visualization [11, 15, 33]; however, this has only been documented anecdotally and has not yet been associated with a clinically significant difference when compared to conventional laparoscopy.

When considering the applicability of gasless laparoscopy to resource-limited settings, a variety of information remains unknown in the literature. While a number of different gasless technology designs have been trialled [14, 19, 34, 35], it is not clear which gasless technology design could be standardized for use in a variety of settings for a variety of procedures. It is also unclear whether trials have been conducted which risk-adjust the complication rates according to patient’s acuity and comorbidities prior to gasless laparoscopy or use compound complication outcomes, and few trials seem to have been performed using randomization [11, 21, 28, 36].

Additionally, it is not clear whether any trials describe the training process for surgeons prior to the trial such as the training that was required to achieve the safety and efficiency outcomes. This is an important factor in implementing a new technique into a rural resource-limited setting which will include the time-investment required for surgeons to learn to use gasless laparoscopy safely and efficiently. To this date, there are no studies for gasless laparoscopy describing the surgical learning curve (i.e., volume of cases required), the ease of adoption into hospitals, and the surgeon and patient satisfaction with their experience with gasless laparoscopy.

This systematic review aims to determine the safety and efficiency of gasless laparoscopy when compared to with alternative treatment methods for surgery.

## Methods/design

### Study design

This systematic review protocol is developed using the Preferred Reporting Items for Systematic Reviews and Meta-analysis Protocol guidelines (PRISMA-P) [37]. The PRISMA statement guidelines including the flowchart will be followed to track all records pulled from all the database search. The study has been registered in the International Prospective Register for Systematic Reviews and Meta-analysis (PROSPERO) with registration number CRD42017078338.

### Search strategy

The study will include an automated and manual search of articles using the databases: MEDLINE (Ovid), Embase (Ovid), Web of Science and Cochrane Central, Global Index Medicus from the World Health Organization (WHO), and the National Institutes of Health clinical trials database. Studies published from 1992 and later will be included which is when gasless laparoscopy was first introduced. The selected articles will then undergo further selection using Boolean terms such as AND/OR. The final included articles will be pooled and managed using Covidence software reference and article manager software. All duplicated will then be identified by the software and removed electronically and manually. The following search terms are used: (((gasless OR non insufflative OR noninsufflative OR non pneumoperiton* OR nonpneumoperiton*).ab,ti OR (abdom* adj3 lift*).ab,ti OR (abdominal wall adj3 (suspension OR elevat*)).ti,ab) AND (laparos* OR laparot* OR peritoneoscop* OR minimally invasive surgery).ab,ti) OR (laparolift OR abdolift).ab,ti. Table 1 shows the search terms used in MEDLINE.

**Table 1:** MEDLINE search strategy

Search number	Search terms	Results
1	(laparolift or abdolift).ab,ti.	21
2	(gasless or non insufflative or noninsufflative or non pneumoperiton* or nonpneumoperiton*).ab,ti.	632
3	(abdom* adj3 lift*).ab,ti.	307
4	(abdominal wall adj3 (suspen* or elevat*)).mp. [mp=title, abstract, original title, name of substance word, subject heading word, floating sub-heading word, keyword heading word, organism supplementary concept word, protocol supplementary concept word, rare disease supplementary concept word, unique identifier, synonyms]	77
5	(laparos* or laparot* or peritoneoscop* or minimally invasive).ab,ti.	209927
6	1 or 2 or 3 or 4	925
7	5 and 6	572

### Study selection

The articles that will be included will follow the PICO tool (Table 2). The population of interest includes human adults of 18 years or more and not restricted to a certain race or geographic location and will include procedures performed at any hospital level. The intervention of interest includes trials involving gasless laparoscopy training, technology or technique, any trial involving general surgical procedures or conditions, or any trial involving diagnostic laparoscopy performed for general surgical investigation, and; any variation of gasless technology and device design, and; any variation of gasless technique, and; any variation of a gasless training program or any mention of background training prior to trial. The comparison group includes patients who have either received no intervention, or; have undergone a general surgical procedure using conventional laparoscopy, or; open laparotomy. The outcomes of interest are the safety and efficiency of gasless laparoscopy. The study will also include case-control, cohort, and randomized control trial and non-randomized control trial studies. In addition, studies published in English or Swedish will be included on or after 1992 (Table 3) and other languages will be excluded, but tracked to understand how many were excluded based on language.

**Table 2:** PICO Tool

Population	All races with no geographical restrictions. Human adults of at least 18 years old will be included from both genders.
Intervention	Gasless laparoscopy
Comparators	Conventional laparoscopy or open laparotomy
Outcomes	Safety and efficiency of gasless laparoscopy

**Table 3:** Inclusion and exclusion criteria

Inclusion criteria	Exclusion criteria
• Articles published in English and Swedish • Articles published on or after 1992 • Adult humans of at least 18 years old of age from both genders with no restrictions to race or geographic distribution • Any trial involving gasless laparoscopy • Studies looking at the safety and efficiency of gasless laparoscopy	• Non-English and non-Swedish published articles • Studies published before 1992 • Patients less than 18 years of age • Non-gasless laparoscopy procedure • Gasless laparoscopy used for gynaecological surgery • *N* < 10 in any arm

### Data extraction and analysis

The review process will include two independent reviewers and then a meeting will be conducted to ensure an overall consensus has been met on all included studies. Should non-agreement on any study arise among the three reviewers, the senior author expertise will be sought to advice on the remaining studies. Screening of studies will start with title and abstract, followed by full-text screening following the inclusion criteria of studies identified a priori. A data extraction spreadsheet will be developed on Microsoft Excel based on variables identified by the research team. The risk of bias across all studies will be assessed using the Cochrane risk of bias assessment tool where the RoB II will be used to assess bias in randomized control trials [38]. The ROBINS-I tool will be used to assess the risk of bias in non-randomized studies [39]. Figures and tables will offer a graphical presentation of the strength of included studies and cumulative risk of bias across all studies. The data will then be analyzed based on the outcomes and subcategories will be identified. The GRADE tool will also be used to help in the full assessment of the quality and risk of bias of the included studies.

### Data synthesis

A qualitative synthesis will be provided for the strength of evidence for each outcome of interest, particularly focusing on difference in harm outcomes between control and intervention groups. This comment will be on the general direction of the effect, and not the effect size, and will focus on results derived from article with low or moderate risk of bias. For each outcome, we will comment on possible precision and strength of association, weakness of existing evidence, sources of systematic bias (limitations, directness, consistency), and overall risk of biases (selection, confounding, performance, attrition, detection and reporting).

A qualitative synthesis will be provided around the strengths and limitations of the included studies, the heterogeneity of existing literature, the generalizability of the synthesized results to other populations, alternative interventions, a variety of settings, and different patient outcomes. Since qualitative synthesis will be sought in this study, the synthesis without meta-analysis (SWiM) reporting guidelines will also be used in conjunction with PRISMA [40]. Additionally, alternate explanations for existing evidence will be considered, and potential for future development may also be discussed. A limitation that could potential arise is the lack of homogeneity of the studies included. This will prompt avoiding meta-analysis of the studies and a qualitative synthesis of the results and description will be used.

## Discussion

The safety of gasless laparoscopy will be reflected through three categories. The first is the number of complications identified from the procedure. Complications might include but are not restricted to pain, hemorrhage, visceral perforation, infection (wound/deep tissue), medical events (pneumonia, acute myocardial infarction, stroke), and biomedical or metabolic effects. The second category includes readmission that can be for a conversion to a laparotomy. The third category is the all-cause mortality from the procedure.

Efficiency of gasless laparoscopy will then be assessed using three categories: the procedural set-up time, the duration of the procedure, and the average length of stay. The results will be discussed in relation to findings of the published literature.

## Data Availability

Not applicable

## Abbreviations

PRISMA-P: Preferred Reporting Items for Systematic review and Meta-Analysis – Protocols
PROSPERO: International Prospective Register for Systematic Reviews and Meta-analysis
WHO: World Health Organization
